# Planning for uncertainty

**DOI:** 10.3389/fpsyg.2012.00541

**Published:** 2012-11-29

**Authors:** Erica C. Yu

**Affiliations:** Decision, Attention, and Memory Lab, Department of Psychology, University of MarylandCollege Park, MD, USA

**A commentary on**

**The nature of belief-directed exploratory choice in human decision-making**

by Knox, W. B., Otto, A. R., Stone, P., and Love, B. C. (2012). Front. Psychology 2:398. doi: 10.3389/fpsyg.2011.00398

With every choice there is a forgone choice, or opportunity cost. When I choose to dine at a particular restaurant, not only am I giving up the opportunity to dine elsewhere, but I am also giving up the opportunity to learn the quality of other restaurants. When the values of alternatives are unknown, over time this dilemma becomes a balance of *exploration* of lesser-known options and *exploitation* of currently favored options.

Knox et al. (2012) investigate exploratory and exploitative behavior to understand whether people update beliefs about the state of their environment in a reflexive way (only in response to observed changes) or a reflective way (without direct observation). This approach for understanding how to learn in complex environments is similar to the dichotomy of model-based and model-free in reinforcement learning (Barto et al., 1990; Sutton and Barto, 1998), which has recently re-emerged in psychology and neuroscience to describe the processes of learning (Daw et al., 2006; Dayan and Daw, 2008; Chater, 2009; Glascher et al., 2010). In modern reinforcement learning, there are typically three levels of learning strategies. In the simplest type, model-free decision makers navigate their environments through simple trial-and-error learning about the value of possible actions based only on direct experiences. In contrast, model-based decision makers use internal representations of the environment to make predictions through simulation in addition to direct experience. And at the third-level, exploration by model-based decision makers can be directed by planning to reduce uncertainty. These strategies map very well to the three models (Naïve RL, Belief model, Ideal Actor model) evaluated by Knox et al. (2012). Although the authors draw upon terminology from social psychology, the findings from this research should broadly inform all research related to learning under uncertainty.

The primary finding from Knox et al. (2012) is that human decision makers learn from interaction with their environment in a reflective manner but do not plan optimally, which is convergent with similar findings by Daw et al. (2006). Decision makers are myopic, in that exploratory choice does not consider the long-term information value of actions. By design, optimal performance in the Leapfrog task requires planning from reflective beliefs: updating beliefs based on past actions and observed payoffs while “considering the effect of exploration on reducing uncertainty in its future beliefs” (Knox et al., 2012, p. 3). In other words, the Ideal Actor places a positive value on exploring for exploration's sake even if he believes it is the inferior payoff option. This may seem to run counter to the goal of the task to choose the superior option as often as possible; however, the rewards for the two actions increase over time but probabilistically alternate in their superiority at an unknown volatility. After a string of exploitative choices, the actor cannot know how many times the state has changed or which option now has the highest value. Given the non-stationary nature of the environment, planning is necessary to succeed in the long run. Exploratory choices have an informational value above and beyond exploitative choices because exploration is the only way by which an actor can learn about the underlying state.

The authors also show that the addition of stochastic choice to the Belief model (i.e., random choice, sometimes choosing the exploratory option even when the actor does not think he should) improves the Belief model's performance on correctly choosing the higher-paying option to nearly the same levels as the Ideal Actor (Table 3 in Knox et al., 2012, p. 8). This simulation begs the follow-up question: if random choice can look like planning, then what does it mean to “plan”? It seems the authors' emphasis on reflexive vs. reflective learning, rather than model-free versus model-based learning, leads to an inability to distinguish between stochasticity and planning. An analysis of the value of planning depends on the actor's prior beliefs about the environment (the actor's model). Indeed, the authors conclude that differing levels of exploration in the Leapfrog task seem best explained by best-fitting *P*(flip) rates (Knox et al., 2012, p. 8) which are likely a consequence of differences in prior beliefs. Including measures of participants' subjective beliefs about *P*(flip) would clarify whether a pattern of choices is myopically stochastic or ideally planned. Using a simple bandit task, Yu and Lagnado (2012) found that subjective beliefs about the probabilities of bandit outcomes influence the structural model that participants generate and use to understand their environment. Knox et al. might find that exploration levels in their task environment likewise depend on participants' prior beliefs. Given the valuable contribution that the authors have made by designing a complex task environment constrained enough to examine optimality, expanding upon the Belief and Ideal Actor models to compare performance based on different prior beliefs about the environment would be a welcome addition to the literature.

Fruitful extensions of this work might focus on incorporating the literature on temporal discounting to build upon the sequential nature of learning in the Leapfrog task. Given that the participants' explicit goal in this task was to maximize the ultimate proportion of correct choices made and yet behavior was sub-optimally myopic, how will participants' behavior change if immediate rewards must compete with distant future rewards (Loewenstein and Elster, 1992; Loewenstein et al., 2003)? Such an environment, analogous to everyday financial, education, and health decisions, may reveal that human decision making is even more dependent on our prior models than the myopic beliefs found in the present study suggest.
